# Seasonal and temporal trends in all-cause and malaria mortality in rural Burkina Faso, 1998–2007

**DOI:** 10.1186/s12936-015-0818-9

**Published:** 2015-08-05

**Authors:** Eveline Otte im Kampe, Olaf Müller, Ali Sie, Heiko Becher

**Affiliations:** Institute of Public Health, University of Heidelberg, Heidelberg, Germany; Centre de Recherche en Santé de Nouna, Nouna, Burkina Faso; Institute for Medical Biometry, University Medical Center Hamburg-Eppendorf, Hamburg, Germany; London School of Hygiene and Tropical Medicine, London, UK

**Keywords:** Malaria, Mortality, Season, Burkina Faso, Cause of death assignment, Sub-Saharan Africa, Time-series-analysis, Fourier-terms

## Abstract

**Background:**

High mortality levels in sub-Saharan Africa are still a major public health problem. Children are the most affected group with malaria as one of the major causes of death in this region. To plan health interventions, reliable empirical information on cause-specific mortality patterns is essential, yet such data are often not available in developing countries. Health and Demographic Surveillance Systems (HDSS) implementing the verbal autopsy (VA) method provide such data on a longitudinal basis. Physician Coded VA is usually used to determine cause of death, but recently a computerized method, Interpreting VA (InterVA) was alternatively introduced. This study investigates the effect of season on all-cause and malaria mortality analysing cause of death data from 1998 to 2007 obtained by the Nouna HDSS in rural Burkina Faso and derived by InterVA.

**Methods:**

Monthly mortality rates were calculated for different age groups (infants, children, adolescents, adults, elderly). Seasonal and temporal trends were modelled with parametric Poisson regression adjusted for sex, area of residence and year of death.

**Results:**

Overall, 7,378 deaths occurred corresponding to a mortality rate of 11.9/1,000 with highest rates in infants (56.8/1,000) and children (22.0/1,000). Young children were most affected by malaria. Malaria mortality patterns in children showed significantly higher rates during the rainy season and a stagnant long-term trend. The seasonal trend is well described parametrically with a sinusoidal function. InterVA assigned about half as many deaths to malaria than physicians.

**Conclusions:**

Malaria mortality remains highly seasonal in rural Burkina Faso. The InterVA method appears to determine reasonably well seasonal mortality patterns, which should be considered for the planning of health resources and activities.

## Background

High mortality levels in sub-Saharan Africa (SSA) are still a major public health problem. This region has the highest rates of childhood mortality with one in eight children facing a probability of dying before age five—that is more than 17 times the average in developed regions [1]. A review of reports from Health and Demographic Surveillance (HDSS) sites in SSA presented pneumonia, diarrhoea and malaria as the main causes of childhood mortality [2], which are preventable and also possible to treat [1]. Another study analysing data from a malaria holoendemic area of north-western Burkina Faso for the time period 1999–2003 showed that malaria was with about 40% the most common cause of death with highest mortality rates among infants aged 6–11 months [3]. Increases in funding and special efforts to fight and prevent malaria in SSA during the last decades have shown critical progress and a widespread reduction of malaria morbidity and mortality [4]. However, about 90% of all malaria deaths worldwide still occur in SSA with children under the age of five as the most affected group [5].

Cause-specific mortality data and information on mortality patterns is very valuable for policy makers to set health priorities for their population and to plan and establish appropriate and proven interventions [6]. Yet, reliable information on the characteristics and health of certain populations is deficient in the developing world [7]. To provide empirical population and health data in developing countries, HDSSs have been established [8] using the verbal autopsy (VA) method to collect cause-specific mortality information in resource-constrained settings [7]. After someone has died, trained field staff conducts an interview with one of the closest relatives of the deceased about signs, symptoms, and circumstances preceding death. However, there might be bias in remembering, reporting and recording such information and the comparability of VA data between different countries is limited by diverse questionnaire designs, different approaches of the interviewers and in interpreting the gathered information although efforts towards standardization have been made [9–11]. The VA method has high rates of sensitivity and specificity for diseases that manifest with a well-defined and unique set of symptoms such as neonatal tetanus, measles, and accidents, but this instrument is less able to discriminate between diseases with overlapping symptoms such as malaria and pneumonia or HIV/AIDS and tuberculosis [12, 13]. It has been shown that VA can overestimate malaria deaths in both low and high transmission settings even where the proportional mortality of malaria among a particular population is very low [14–16].

However, despite obvious limitations the VA method is at present the principle way to study cause of death (COD) information in the developing world and has consequently been applied in a number of countries [17].

Later, VA data can be interpreted to identify a COD [18] by different methods, such as physician review and interpretation of verbal autopsy (InterVA) [19]. Physician-certified verbal autopsy (PCVA) is still the most common approach to interpret VA data [20] in which local physicians review the questionnaires and assign a probable COD [16, 21–24]. Arguments against physician-based certification of CODs from VAs are variations in interpreting VA data due to different training, experience, and/or perceptions of local epidemiology of the reviewing physicians and difficulties of maintaining quality work over long periods of time [25].

A new method to interpret VA data is a computerized model known as InterVA (Interpreting Verbal Autopsy). Although InterVA relies on symptom reports for its algorithmic approach to assigning cause of death it is a more consistent approach than PCVA [26]. Based on Bayes’ probability theorem the InterVA model weights symptoms reported during the VA process in relation to specific CODs and determines up to three probable CODs and their corresponding likelihoods [27].

Despite the considerable number of studies analysing mortality patterns in SSA [2–4, 17, 28, 29], relatively few have set their focus on seasonal patterns [3, 30–36]. An analysis of HDSS data on CODs determined by PCVA from a malaria holoendemic region in Burkina Faso investigated seasonal patterns of malaria mortality for the period 1999–2003 [33] and found consistently higher childhood mortality during and at the end of the wet season, when transmission intensity of malaria is at its highest. Since that study used COD data interpreted by physicians only who are known to overdiagnose malaria in holoendemic regions [37, 38], the aim of this study is to investigate the impact of season on all-cause and malaria mortality in different age groups using InterVA COD data. In addition, a larger observation period including more recent data was analysed for the present study taking into account the effect of year on mortality, which was not done in the previous analysis.

## Methods

### Study population

This study is based on data from the Nouna HDSS which had 80,000 individuals at the end of 2007. It is run by the Centre de Recherche en Santé de Nouna [39] and located in the North-West of Burkina Faso in a rural area predominated by a sub-Sahelian climate with one rainy (June–October) and dry (November–May) season per year [3, 40]. This study covers the observation period from 1 January 1998 to 31 December 2007. Data for Nouna town could only be analysed since 1 January 2000 after Nouna town was integrated into the study area.

To record COD data, the Nouna HDSS has been applying the VA method since 1993. After a death has occurred, trained interviewers visit the household of deceased people in the study area to conduct the VA interview using a standardized questionnaire after obtaining oral informed consent. Most VA interviews are carried out between 3 and 6 months after death allowing for the mourning period [41].

For this study, the InterVA-3 method was applied here to derive the most likely cause of 35 possible COD groups [42]. A detailed description of the InterVA model has been given elsewhere [43]. In short, the InterVA model defines the probability of a cause for a particular death given the presence of a specific disease indicator or symptom using an automated Bayesian model [26]. It displays up to three probable CODs and their corresponding likelihoods. In order to consider local epidemiology for important diseases in the Nouna HDSS region, the malaria and HIV/AIDS prevalence was set to “high” for malaria and to “low” for HIV/AIDS [44]. Results will be compared with the previously most often used method to derive the cause of death from VA data, i.e. physician coding.

### Data analysis

All individuals registered in the Nouna HDSS within the study period were included in the analysis except a few individuals (N = 97) for whom no information on month of death were available. To calculate monthly mortality rates, the population by month, year, sex, age group, and area (Nouna town and rural) was estimated as the average of the population at the beginning and end of a month (mid-month-population). Age groups were defined as follows: infants (<1 year), children (1 to <5 years), adolescents (5 to <15 years), adults (15 to <60 years) and the elderly (60+ years). Likewise, the total number of deaths in these categories was calculated. For malaria-specific analysis, the groups “malaria”, “other causes”, and a third category containing missing causes either due to missing data or causes that could not be determined by either method (PCVA or InterVA) were considered. Only CODs with the highest likelihood as estimated by InterVA, were considered as COD for an individual in the analysis.

The monthly mortality rates μ per 1,000 were calculated as μ = (D/M) × 1,000 in which D denotes the number of deaths in the respective month. M denotes the approximate estimate of the person-years, estimated by dividing the mid-month-population by 12.

To analyse malaria mortality, an imputation procedure was used to consider deaths with missing verbal autopsy questionnaire. The probability that a death with missing questionnaire was due to malaria was estimated by a log-linear model depending on age group and month. The number of deaths with missing VA questionnaire, multiplied with this probability is then the expected number of additional malaria deaths in a given month and age group.

For graphical assessment of seasonal variations and long-term trends, a weighted 5-month moving average (MA) was used according to

$${\rm M}{\rm A}_{\text{month}} = \,0.4 \, \times \, \upmu_{\text{month}} \,\,+\,\, 0.2 \, \times \, (\upmu_{{{\text{month}}+1}} + \upmu_{{{\text{month}}-1}} ) \,\, + \,\, 0.1 \, \times \, (\upmu_{{{\text{month}}+2}} \,\, + \,\, \upmu_{{{\text{month}}-2}} ).$$

For assessing the relative monthly effect on overall and malaria mortality, age group-specific Poisson regression models were fitted according to

- Model I
  $$\ln \, [\upmu \left( {{\text{x}}_{ 1} , {\text{ x}}_{ 2} , {\text{ x}}_{ 3} , {\text{ x}}_{ 4} } \right)] = \upbeta_{1} {\text{x}}_{1} + \upbeta_{2} {\text{x}}_{ 2} + \upbeta_{3} {\text{x}}_{ 3} + \upbeta_{4} {\text{x}}_{4})$$
  and
- Model II
  $$\ln \, [\upmu \left( {{\text{x}}_{ 2} , {\text{ x}}_{ 3} , {\text{ x}}_{ 4} } \right)] = \upbeta_{0} + \upbeta_{2} {\text{x}}_{ 2} + \upbeta_{3} {\text{x}}_{ 3} + \upbeta_{4} {\text{x}}_{ 4}$$

Model I is defined as a model without intercept, where x_1_ is a vector with binary dummy variables for each month, x_2_ is a continuous variable for calendar year running from 1 (year 1998) to 10 (year 2007) to investigate an overall change in rates over time, x_3_ represents sex and x_4_ area. Model I has no intercept and calculates an estimate for each month. In Model II, an intercept β_0_ instead of a monthly effect is estimated. The relative monthly effect on mortality was calculated by the difference β_1_–β_0_ of the monthly effect of Model I and the overall effect of model II.

To further assess the seasonal effect on malaria mortality, rate rations (RRs) were estimated using a Poisson regression model with a continuous function of month of death. For this, a sine-function was used of the form g_1_(x_1_) = sin (x_1_ × π/6) and a cosine-function of the form g_2_(x_1_) = cos (x_1_ × π/6) in which x_1_ adopts a value between 1 and 12, corresponding to the months January to December. This resulted in the model

- Model III
  $$\ln \, [\upmu \left( {{\text{x}}_{ 1} , {\text{ x}}_{ 2} , {\text{ x}}_{ 3} , {\text{ x}}_{4} } \right)] = \upbeta_{0} + \upbeta_{11} g_{1} \left( {{\text{x}}_{1} } \right) + \upbeta_{12} g_{2} \left( {{\text{x}}_{1} } \right) + \upbeta_{2} {\text{x}}_{2} + \upbeta_{3} {\text{x}}_{3} + \upbeta_{4} {\text{x}}_{4}.$$

From the regression parameters β_11_ and β_12_ the amplitude is calculated as $\sqrt {\beta_{11}^{2} + \beta_{12}^{2} }$ and the phase φ as arctan(−β_11_/β_12_) which determines the day of the year with the highest rate. To test the strength of evidence, the difference of deviances of Model II and III was calculated which is asymptotically χ2-distributed with two degrees of freedom since two parameters (β_11_, β_12_) are estimated. Effects are calculated as logarithmic RR.

Since Nouna town was encompassed in the study area in 2000, 432 out of 480 observations, determined by all possible cross-classifications of the variables year, sex and area for which people were observed, were included in each model. For every model 48 observations were set missing, because the number of individuals in these observations was zero. Data analysis was carried out with SAS, 9.2. Poisson regression used the SAS-procedure PROC GENMOD.

## Results

During the whole study period, 7,378 deaths occurred, corresponding to a crude mortality rate of 11.9/1,000 (95% CI 11.7–12.2). From these, a total of 5,621 (76.2%) VA questionnaires were completed. Table 1 shows the numbers of missing VAs with highest proportions for infants and young people. The average proportion of missing VAs was 23.8% and continuously decreased over the time of the observation period.

**Table 1:** Deaths, missing verbal autopsy (VA) questionnaires and missing causes due to no consensus between physicians, undetermined and ill-defined causes by age group, Nouna HDSS, 1998–2007

	Infants (<1)	Children (1–4)	Adolescents (5–14)	Adults (15–59)	Elderly (60+)	Total
Deaths	1,436	1,927	452	1,580	1,983	7,378
Missing VAs	384	443	116	388	426	1,757
Missing VAs (%)	26.7	23.0	26.7	24.6	12.4	23.8
No consensus	64	98	25	125	174	486
No consensus (%)	4.5	5.1	5.5	7.9	8.8	6.6
Ill-defined	55	65	45	183	295	643
Ill-defined (%)	3.8	3.4	10.0	11.6	14.9	8.7
Undetermined	86	112	35	105	160	498
Undetermined (%)	6.0	5.8	7.7	6.6	8.1	6.7

Usually, if two physicians cannot agree on a COD, a third physician reviews the VA questionnaire to determine a COD. However, if a third physician was not available due to constraint resources or all three physicians determined a different COD, this might result in additional missing CODs. Numbers for these PCVA missings were very similar for the first three age groups but increased for adults and old people. Numbers of ill-defined PCVA CODs resulting from insufficient or unclear information were relatively small for children under age five but increased for the older age groups. The “undetermined” category among the InterVA CODs encompasses all deaths, which could not be determined by the InterVA model. It is larger for infants and children (~6%) than in the ill-defined category determined by PCVA. For the older age groups, an opposite picture is shown. Here, the percentage of undetermined causes is smaller than in the ill-defined category.

The all-cause mortality rate over the whole study period was 56.8/1,000 (95% CI 53.9–59.8) for infants, 22.0/1,000 (95% CI 21.0–23.0) for children, 2.5/1,000 (95% CI 2.3–2.7) for adolescents, 5.4/1,000 (95% CI 5.2–5.7) for adults and 56.3/1,000 (95% CI 53.8–58.8) for the elderly (see Table 2). According to the InterVA coding method, high malaria mortality rates were found only for infants (8.0/1,000; 95% CI 6.9–9.1). This is in contrast to malaria mortality estimates of CODs determined by PCVA showing relatively high malaria mortality also among children and the elderly with rates of 9.4/1,000 (95% CI 8.7–10.0) and 8.1/1,000 (95% CI 7.2–9.1), respectively. Overall, significantly less malaria causes were determined by InterVA than by PCVA except for adults. Here, the malaria mortality rate was with 0.4/1,000 (95% CI 0.3–0.4) for PCVA significantly lower in comparison to InterVA with 0.6/1,000 (95% CI 0.5–0.7). For adults, both methods showed that the proportion of malaria was the smallest and deaths assigned to the “other causes” category accounted for more than half of all adult deaths. A full analysis of COD is not intended here.

**Table 2:** Mortality by age and coding method, Nouna HDSS, 1998–2007 (rates per 1,000)

	Infants (<1)	Children (1–4)	Adolescents (5–14)	Adults (15–59)	Elderly (60+)	Total
PCVA						
Malaria						
N	524	823	105	108	286	1,846
Rate	20.7	9.4	0.6	0.4	8.1	3.0
95% CI	19.0–22.5	8.7–10.0	0.5–0.7	0.3–0.4	7.2–9.1	2.8–3.1
%	36.5	42.7	23.2	6.8	14.4	25.0
Other causes						
N	458	563	178	831	875	2,905
Rate	18.1	6.4	1.0	2.9	24.8	4.7
95% CI	16.5–19.8	5.9–7.0	0.8–1.1	2.7–3.1	23.2–26.5	4.5–4.9
%	31.9	29.2	39.4	52.6	44.1	39.4
Ill-defined + missing						
N	454	541	169	641	822	2,627
Rate	18.0	6.2	0.9	2.2	23.3	4.2
95% CI	16.3–19.6	5.7–6.7	0.8–1.1	2.0–2.4	21.7–24.9	4.1–4.4
%	31.6	28.1	37.4	40.6	41.5	35.6
Total						
N	1,436	1,927	452	1,580	1,983	7,378
Rate	56.8	22.0	2.5	5.4	56.3	11.9
95% CI	53.9–59.8	21.0–23.0	2.3–2.7	5.2–5.7	53.8–58.8	11.7–12.2
InterVA						
Malaria						
N	203	342	49	178	53	825
Rate	8.0	3.9	0.3	0.6	1.5	1.3
95% CI	6.9–9.1	3.5–4.3	0.2–0.3	0.5–0.7	1.1–1.9	1.2–1.4
%	14.1	17.7	10.9	11.3	2.7	11.2
Other causes						
N	806	1,103	268	959	1,421	4,557
Rate	31.9	12.6	1.5	3.3	40.3	7.4
95% CI	29.7–34.1	11.8–13.3	1.3–1.7	3.1–3.5	38.2–42.4	7.2–7.6
%	56.1	57.2	59.3	60.7	71.7	61.8
Undetermined + missing						
N	421	490	134	438	513	1,996
Rate	16.7	5.6	0.7	1.5	14.5	3.2
95% CI	15.1–18.3	5.1–6.1	0.6–0.9	1.4–1.7	13.3–15.8	3.1–3.4
%	29.3	25.4	29.6	27.7	25.9	27.1

**Fig. 1:**
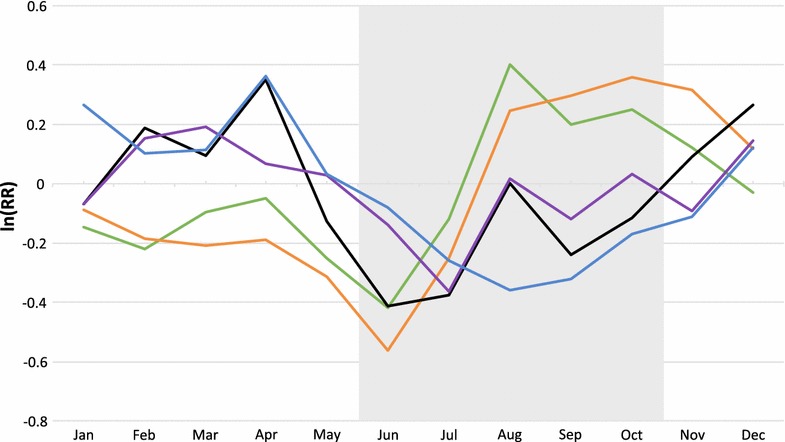
Relative effect of month of death by age group, Nouna HDSS 1998–2007. Infants <1 (*green*), children 1–4 (*orange*), adolescents 5–14 (*black*), adults 15–59 (*purple*), elderly 60+ (*blue*), *grey shaded* area (rainy season).

Figure 1 presents the age-specific relative monthly effect on all-cause mortality. For infants and children, the logarithmic RRs were higher in the months of the wet season, whereas old people showed an opposite trend. The relative monthly effect for youth and adults is similar to the trend for the oldest, but the picture is less clear. For all age groups, the variation of the monthly rates was significant.

**Fig. 2:**
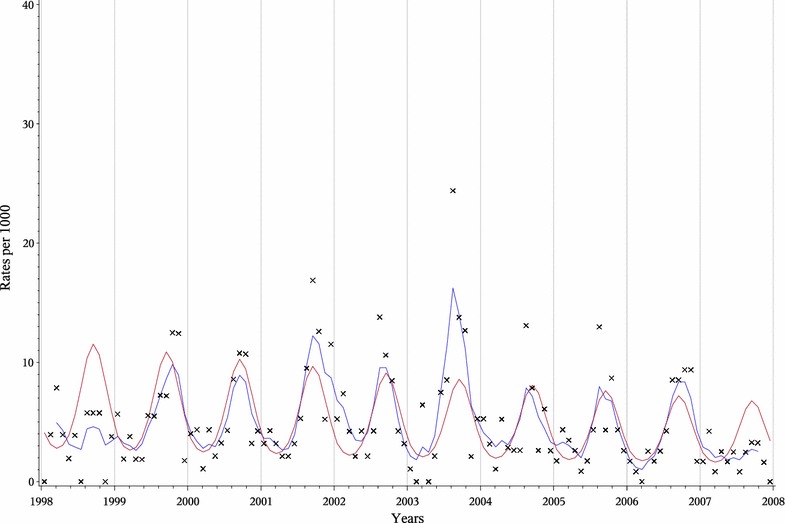
Malaria mortality rates according to InterVA for children under five, Nouna HDSS, 1998–2007. Moving average (*blue*), model (*red*), original rates (*X*).

In Fig. 2 the modelled monthly mortality rates and the MA of the rates are illustrated for malaria diagnoses based on InterVA over the whole study period. Infants and children were combined in this analysis. A remarkable agreement was observed between the model-based estimates (in red) and the moving average estimates (in blue). A slight decreasing trend of malaria mortality was observed over the whole study period which is however highly significant (p = 0.0001).

## Discussion

This study adds further evidence to a continuously huge impact of malaria on mortality in young children of rural malaria endemic regions of SSA [3, 17, 45–50]. While a large proportion of deaths in infants were attributed to malaria (20.7/1,000; 95% CI 19.0–22.5) by physicians’ diagnosis, InterVA attributed much less of all infant deaths to malaria. A similar pattern occurred for diagnoses in adolescents, adults and the elderly, but with a much lower proportion of deaths attributed to malaria. In the elderly, physicians attributed a much higher proportion of deaths to malaria compared to InterVA [17], which is likely based on the assumption of a weakening immune system among the elderly [40, 51, 52]. Overall, physicians assigned more deaths to malaria than InterVA did, clearly due to physicians’ tendency to overdiagnose malaria in malaria-endemic areas [37, 38]. An exception could be seen in female adults. Most non-pregnant women in SSA have been exposed to falciparum malaria and are semi-immune to this disease [53]. Local physicians might expect pregnant women living in malaria holoendemic regions to be semi-immune and tend to diagnose other infections than malaria. Given that most adult women in this study were of reproductive age (15–49) this might explain the higher malaria mortality levels according to InterVA diagnoses in comparison to PCVA. However, it cannot fully be judged which method is closer to the real values.

One limitation of this study is the high proportion of unknown CODs due to missing VA questionnaires or insufficient data resulting in assignment of deaths to the “ill-defined”/“undetermined” category. It is very likely that among deaths with unknown cause some were due to malaria. The imputation method used in the analysis was based on the reasonable assumption that deaths with unavailable verbal autopsy information were equally likely to have died of malaria as cases with VA information. If all these deaths were considered as causes other than malaria, the results would have been similar, however the amplitude in Fig. 2 would be about twenty percent lower.

Moreover, there are well-known limitations of the VA method itself relating to the validity of CODs, especially for diseases with non-specific symptoms like malaria. Overlapping symptoms between malaria and diseases such as acute respiratory infection may lead to misclassification of diagnoses and affect the accuracy of the results. Such inaccuracies might additionally be due to variations in VA sensitivity for malaria deaths, depending in areas of high transmission intensity more on the incidence and prevalence of malaria than on exact diagnostic definition [47]. The decrease in VA specificity for malaria with increasing malaria proportional mortality [47] is accompanied by lower sensitivity of VA for other CODs in sites with intense malaria transmission [54]. Thus, use of VA data makes it difficult to estimate the entire impact of malaria on mortality in high transmission settings due to lower specificity and sensitivity. Performance of VA in determining CODs may be better in areas where malaria play only a minor role.

Another limitation of this study is that only one COD with the highest likelihood as displayed by the InterVA output was included into the analysis, possibly leading to a distortion of the estimates among InterVA causes. However, the overall pattern of CODs did not change when comparing the weighted InterVA approach to the one considering only the most likely COD. Unfortunately, no gold standard is available yet against which both methods could be validated. Thus, the true CODs are impossible to obtain [55] and uncertainty levels with either procedure are high [44] in particular for malaria, which is difficult to diagnose accurately without parasitic evidence [56, 57]. Hence, mortality statistics based on VA have to be interpreted with caution.

This study also provides additional evidence of the seasonal effect on mortality during the rainy season in this part of SSA, which is mostly attributable to malaria [33, 35, 58]. This trend was presented in the regression model with a peak in the wet season, which can be modelled by a parametric sinusoidal curve. The validity of the modelling approach was checked graphically by comparison to the MA of the rates, which were quite similar to the modelled rates. A previous study on seasonal mortality patterns showed that this is an appropriate approach to investigate seasonal mortality patterns [33]. Overall, the mortality rates among the different COD categories showed discrepancies between InterVA and PCVA. The estimated malaria mortality was considerably higher (20.7/1,000) among physicians’ diagnoses.

## Conclusions

The present study supports a continuous marked seasonality of malaria mortality in the endemic regions of rural West Africa. Furthermore, it confirms the high impact of malaria on mortality in young children in rural Burkina Faso with excess at around the end of the rainy season.

However, data for the present study was only available up until 2007 and several malaria control interventions have been conducted in the study area afterwards such as the distribution of insecticide-treated nets (ITN) that increased the coverage of at least 1 ITN per household to 99% in 2011 [59]. Furthermore, artemisinin-based combination therapy (ACT) became available in all Centres de Santé et Promotion Sociale in late 2007 [60]. However, only 27% of all available ITNs were used and only about half of those were used by either under five children or pregnant women. Also, a large proportion of children were treated with inadequate medications. Thus, it is still important to support and improve malaria programs in SSA.

Moreover, it was shown that the probabilistic InterVA model determines reasonably well seasonal patterns of mortality. Such information will be useful for the planning of health resources and activities in resource-limited settings where reliable estimates of morbidity and mortality are lacking.
